# Anticancer prodrugs of butyric acid and formaldehyde protect against doxorubicin-induced cardiotoxicity

**DOI:** 10.1038/sj.bjc.6603781

**Published:** 2007-05-01

**Authors:** A Rephaeli, S Waks-Yona, A Nudelman, I Tarasenko, N Tarasenko, D R Phillips, S M Cutts, G Kessler-Icekson

**Affiliations:** 1 Sackler Faculty of Medicine, Felsenstein Medical Research Center, Tel-Aviv University, Beilinson Campus, Petach-Tikva, 49100, Israel; 2 Chemistry Department, Bar-Ilan University, Ramat-Gan, 52900, Israel; 3 Department of Biochemistry, La Trobe University, Victoria 3086, Australia

**Keywords:** doxorubicin, cardiomyocytes, formaldehyde, prodrugs, histone acetylation

## Abstract

Formaldehyde has been previously shown to play a dominant role in promoting synergy between doxorubicin (Dox) and formaldehyde-releasing butyric acid (BA) prodrugs in killing cancer cells. In this work, we report that these prodrugs also protect neonatal rat cardiomyocytes and adult mice against toxicity elicited by Dox. In cardiomyocytes treated with Dox, the formaldehyde releasing prodrugs butyroyloxymethyl diethylphosphate (AN-7) and butyroyloxymethyl butyrate (AN-1), but not the corresponding acetaldehyde-releasing butyroyloxydiethyl phosphate (AN-88) or butyroyloxyethyl butyrate (AN-11), reduced lactate dehydrogenase leakage, prevented loss of mitochondrial membrane potential (Δ*Ψ*m) and attenuated upregulation of the proapoptotic gene Bax. In Dox-treated mice, AN-7 but not AN-88 attenuated weight-loss and mortality, and increase in serum lactate dehydrogenase. These findings show that BA prodrugs that release formaldehyde and augment Dox anticancer activity also protect against Dox cardiotoxicity. Based on these observations, clinical applications of these prodrugs for patients treated with Dox warrant further investigation.

Cardiotoxicity is a significant complication of cancer treatment. The anthracycline doxorubicin (Dox) is one of the most widely employed anticancer agents whose clinical use is limited by acute and chronic cardiotoxicity (DeVita *et al*, 2001). Studies have shown that heart failure in Dox-treated patients may take place many years after treatment cessation (Steinherz and Steinherz, 1991). Retrospective analysis of three clinical trials with 630 breast carcinoma and small-cell lung carcinoma patients indicated that at a cumulative dose of 550 mg m^−2^, an estimated 26% of patients experienced Dox-related congestive heart failure (Swain *et al*, 2003).

The molecular basis of Dox-induced cardiotoxicity is attributed to several different molecular events: generation of reactive oxygen species (ROS), impaired calcium homeostasis and mitochondrial functions, reduced expression of energy-generating enzymes and degradation of Dox to its toxic metabolite doxorubicinol (Mohamed *et al*, 2000; Minotti *et al*, 2001). The vulnerability of the heart to ROS is further intensified by Dox inhibition of ROS neutralising enzymes (Li *et al*, 2000). The drug ICRF-187 (dexrazoxane) is the only registered and clinically successful cardiotoxicity modulator that chelates iron and inhibits formation of the oxygen-free radicals, yet the controversy about its effect against cardiotoxicity justifies a search for additional protective agents (Hofland *et al*, 2005; Elbl *et al*, 2006).

We have previously studied a family of acyloxyalkyl ester prodrugs of histone deacetylase (HDAC) inhibitors having the general formula Me(CH_2_)_2_COOCH(R)OR^1^, (R=H, Me, Pr, *t*-Bu; R^1^=OC-alkyl, and P(O)(OEt)_2_) that upon metabolic hydrolysis release acids and aldehydes. These prodrugs possess HDAC inhibitory activity, anticancer activity and low toxicity (Nudelman *et al*, 2005; Rephaeli *et al*, 2006). The most potent ones release butyric acid (BA) and formaldehyde upon metabolic hydrolysis (Nudelman *et al*, 2005). As anticancer agents they act in synergy with Dox by increasing the number of Dox–DNA adducts formed (Cutts *et al*, 2001; Engel *et al*, 2006; Swift *et al*, 2006).

Recent studies have attributed to histone acetylation a crucial role in regulating both pro- and antihypertrophic pathways in the heart (Zhang *et al*, 2002; Antos *et al*, 2003; Kook *et al*, 2003). Histone deacetylase-9 has been shown to act as a signal-responsive repressor of cardiac hypertrophy (Zhang *et al*, 2002). Consistent with this repressive role, mutant mice lacking HDAC9 are hypersensitive to hypertrophic signals. In contrast, HDAC2 has been found in a functional complex with the homeobox protein Hop, which stimulates hypertrophy by blocking an antihypertrophic gene programme dependent on serum response factor (Kook *et al*, 2003). Histone deacetylase inhibition *in vivo* and *in vitro* antagonised hypertrophic stimulation of myocytes, supporting the notion of histone hyperacetylation-dependent signalling of cardiac hypertrophy (Antos *et al*, 2003).

In light of the synergistic interaction between BA and formaldehyde-releasing acyloxyalkyl prodrugs and Dox in killing cancer cells, we investigated the manner by which they affect Dox-treated cardiomyocytes and adult mice and found that they protect against Dox toxicity *in vitro* and *in vivo*. These observations make the acyloxymethyl ester prodrugs and particularly AN-7, attractive candidate drugs for cancer treatment.

## MATERIALS AND METHODS

The study was conducted according to the NIH Laboratory Animal Care Guidelines approved by the Tel Aviv University Committee for Animal Experimentation.

### Compounds and reagents

The structure and hydrolysis products of the BA prodrugs, synthesised as described (Nudelman *et al*, 2001) and used in this study, are presented in Table 1. For *in vitro* studies, AN-7 was dissolved in the indicated growth medium whereas AN-88, AN-1 and AN-11 were dissolved in DMSO and diluted in the growth medium to the desired concentration. The final DMSO concentration was ⩽0.1%. Solutions of the prodrugs were handled using Hamilton syringes and glass vials. For *in vivo* studies, AN-7 was dissolved in saline, and AN-88 in soy oil.

Tissue culture media and serum were obtained from Biological Industries, Beit-Haemek (Israel); Dox hydrochloride, 2 mg ml^−1^ was from Teva (Petach Tikva, Israel), and JC1 (5,5′,6,6′-tetrachloro-1,1′,3,3′-tetraethylbenzimidazolylcarbocyanine iodide) was from Alexis (San Diego, CA, USA). All other chemicals used, if not specifically indicated, were purchased from Sigma, St Louis, MO, USA.

### Antibodies

Primary antibodies for Western blot analysis: rabbit anti-acetylated H4 (lysine 12) and rabbit anti-histone H3 were from Cell Signaling (San Diego, CA, USA). Secondary antibodies: horseradish peroxidase (HRP)-goat anti-rabbit IgG and HRP-goat antimouse IgG were from Jackson ImmunoResearch (West Grove, PA, USA).

### Cardiomyocyte culture

Neonatal rat ventricular myocytes were isolated and cultured in collagen-coated culture dishes as described (Shalitin *et al*, 1996). Growth medium contained DMEM:Ham's F12 (1 : 1), fetal calf serum (10%) and antibiotics. Treatment was administered to spontaneously beating cardiomyocytes 48 h after plating.

### Cytotoxicity evaluation

Cardiomyocytes were seeded in 24-well plates (5 × 10^5^  cells 500 *μ*l^−1^ well^−1^) and 2 days later were treated as follows: the prodrugs (Table 1) were added to the culture either alone for 24 or 1 h before addition of 2 *μ*M Dox. After 5 h, the medium containing Dox was removed and the prodrug-containing medium was added again for a total exposure time of 24 h. Plates were centrifuged for 5 min at 700 **g**, the supernatant was removed and lactate dehydrogenase (LDH) activity was determined in the growth medium and in the remaining cell layer.

### Lactate dehydrogenase assay

Lactate dehydrogenase activity was used to assess damage induced by Dox. Measurements of LDH released into the culture medium or that which remained in the cell layer, were carried out spectroscopically with CytoTox 96 non-radioactive cytotoxicity assay (Promega, Madison, WI, USA). Control (100%) was defined as LDH released from untreated cells. To optimise the assay we validated that increased LDH in the medium correlated with decreased LDH activity in the cell layer.

### Assessment of changes in mitochondrial membrane potential (Δ*ψ*m)

The fluorescent mitochondrial-specific cationic dye JC-1 was used to assess the status of Δ*ψ*m in cardiomyocytes after drug treatment. JC-1 undergoes potential-dependent accumulation in the mitochondria. Mitochondria with a normal Δ*ψ*m concentrate aggregated JC-1 (red fluorescence) and in depolarised mitochondria, JC-1 forms monomers (green fluorescence). Rat neonatal cardiomyocytes were seeded in 24-well plates (6 × 10^5^ cells 500 *μ*l^−1^ well^−1^) and treated with 2 *μ*M Dox for 5 h, 100 *μ*M AN-7, AN-88, or the combination of Dox with either AN-7 or AN-88. The prodrug was added 1 h before the addition of Dox for 5 h. The Dox was washed out and medium containing the prodrug was added to the cells for an additional 18 h. A stock solution of JC-1 (1 mM in DMSO) was added to 40 mM HEPES buffer, pH 7.4, supplemented with 0.65% NaCl and 4.5 g l^−1^ glucose at 37°C. The final concentration of JC-1 was 10 *μ*M and was loaded into the mitochondria by incubation of the cells for 60 min at 37°C. Following the incubation, the cells were washed with HEPES buffer (500 *μ*l) and images of the stained cells were examined under a fluorescent microscope (IX70; Olympus Tokyo, Japan) using excitation filter of 330–385 nm and barrier filter at 420 nm. Photographs were captured with an Olympus DP50 digital camera system. Two independent experiments performed in triplicates were photographed, and the images were analysed by the ImagePro Plus 5.1 software. The ratio of green to red areas was measured in >6 different fields in each experimental group and the mean±s.e.m. calculated.

### RNA isolation and analysis

Cells were seeded in 60-mm culture dishes treated with drugs, and total RNA was extracted using guanidinium isothiocyanate as described (Chomczynski and Sacchi, 1987), quantified spectrophotometrically and its integrity validated by electrophoresis in 1% agarose gels (Shalitin *et al*, 1996). A total of 1 *μ*g of total RNA was reverse-transcribed using M-MLVRT(H-) (Promega) and oligo dT (10 *μ*M). For semiquantitative RT–PCR, the resulting cDNA samples were amplified with the indicated primer pairs under the corresponding precalibrated conditions specified in Table 2. For product amplification, we employed the TaKaRa *Taq* polymerase (TaKaRa, Shiga, Japan), except for the Bax amplification with primer pair [I] where the *Taq*Hot (New England Biolabs, Beverly, MA, USA) was used. The cycling protocol was 30 s denaturation at 95°C, 30 s annealing at the indicated temperature (Table 2), 50 s elongation at 72°C. The PCR products were resolved in 2% agarose gels, visualised by ethidium bromide staining and UV translumination, and quantified in the VersaDoc Imaging System using Quantity One Software (Bio-Rad Laboratories, Hercules, CA, USA). Expression levels were normalised to that of GAPDH mRNA in each sample, and the fold change in expression was calculated relative to the untreated control cultures.

### Histone acetylation in cardiomyocytes

Cells seeded in 100-mm culture dishes and at 70–80% confluency were treated with AN-7 (100 *μ*M) for 4 h. The medium was discarded and the cells were rinsed with ice-cold PBS and harvested with a rubber policeman. For histone determination, cells were lysed in 1 ml of lysis buffer (10 mM HEPES, pH 7.9, 1.5 mM MgCl_2_, 10 mM KCl, 0.5 mM DTT, 1.5 mM phenylmethanesulfonyl fluoride) and histones were extracted and subjected to Western blot analysis as described (Nudelman *et al*, 2005).

### Histone acetylation *in vivo*

For determination of histone acetylation *in vivo*, female C57/BL mice were treated orally with AN-7 at a dose of 100 mg kg^−1^ for 30 min, 5 h and 24 h. The heart, liver and kidneys were removed, frozen immediately in liquid nitrogen and kept at −70°C. Histones were purified and subjected to Western blot analysis (Nudelman *et al*, 2005; Rephaeli *et al*, 2006).

### Doxorubicin-induced toxicity *in vivo*

Female C57/BL mice, 6–10 weeks old (Harlan, Rehovot, Israel) were treated with a single i.p. dose of 20 mg kg^−1^ Dox and three times a week p.o. with vehicle, AN-7 (20 mg kg^−1^) or AN-88 (20 mg kg^−1^) for 2 weeks. AN-7 and AN-88 were given 1 h before Dox. At the end of the experiment, blood was drawn from the eyes, allowed to coagulate, centrifuged twice (10 min, 778 **g**) and the collected serum was analysed for LDH with CytoTox 96 non-radioactive cytotoxicity assay (Promega). Control (100%) was defined as the LDH activity in untreated mice.

### Statistical analysis

Statistical analysis was performed using Excel Office 2000 for Student's *t*-test, linear regression test and *χ*^2^ test.

## RESULTS

### Protection of neonatal cardiomyocytes against Dox-induced toxicity

**Figure 1:**
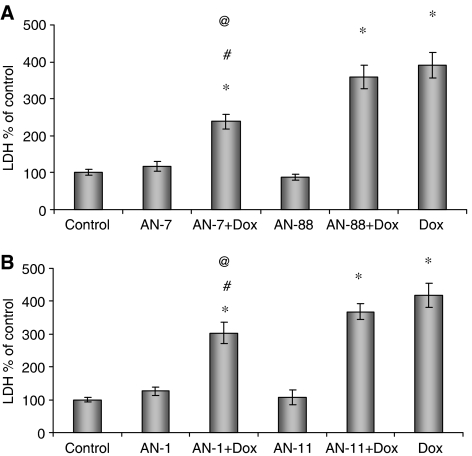
Protection against Dox-induced toxicity in cardiomyocytes. Cardiomyocytes were seeded in 24-well plates (5 × 10^5^ cells well^−1^) and 2 days later treated with (**A**) 100 *μ*M of AN-7 or AN-88, 2 *μ*M Dox, the combinations of 2 *μ*M Dox and 100 *μ*M AN-7, 2 *μ*M Dox and 100 *μ*M AN-88 or none (control); (**B**) 100 *μ*M AN-1 or AN-11, 2 *μ*M Dox, the combinations of 2 *μ*M Dox and 100 *μ*M AN-1, 2 *μ*M Dox and 100 *μ*M AN-11 or none (control). Lactate dehydrogenase activity in the growth medium was measured after 24 h as specified in Materials and Methods. Data summarise four (**A**) and three (**B**) independent experiments. ^*^P<0.05 *vs* control, ^#^*P*<0.05 *vs* Dox, ^@^*P*<0.05 AN-7+Dox *vs* AN-88+Dox or ^@^*P*<0.05 AN-1+Dox *vs* AN-11+Dox.

The prodrugs listed in Table 1 were examined for their ability to protect cardiomyocytes from Dox-induced toxicity. Their effect on LDH release from the myocytes to the growth medium (after 24 h of treatment with Dox) was measured (Figure 1). In preliminary dose–response experiments, a concentration of 2 *μ*M Dox was found to cause maximal damage while the prodrugs alone, up to 200 *μ*M, did not induce damage to the cells (data not shown). Moreover, the Dox-induced damage was significantly reduced when co-treated with prodrugs releasing formaldehyde and BA (AN-7, AN-1). This protective effect was not observed after treatment with the combination of Dox and analogous prodrugs that release acetaldehyde and BA (AN-88, AN-11). The results indicate that formaldehyde plays a central role in protecting the cardiomyocytes against Dox-induced damage. It is important to note that the effect of prodrugs that release one equivalent of BA (AN-7 or AN-88) was similar to that of prodrugs that release two equivalents of BA (AN-1 or AN-11, respectively), indicating that the number of BA equivalents released by the prodrugs does not contribute to the protective activity.

### Protection of neonatal cardiomyocytes from Dox-induced dissipation of mitochondrial membrane potential

**Figure 2:**
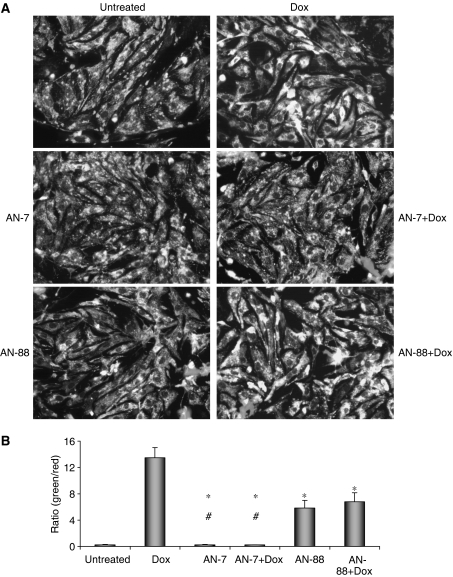
The effect of AN-7 and Dox on mitochondrial membrane potential in neonatal rat cardiomyocytes. Cardiomyocytes (6 × 10^5^ cells well^−1^, 24-well plates) were grown for 72 h and then treated with 2 *μ*M Dox for 5 h, 100 *μ*M AN-7, 100 *μ*M AN-88 or the combination as indicated. In the combination, the prodrug was added 1 h before the addition of 2 *μ*M Dox for 5 h, when Dox was washed out and medium containing 100 *μ*M AN-7 or 100 *μ*M AN-88 was added for additional 18 h. The cells were stained with 10 *μ*M JC-1 for 1 h at 37°C and photographed at a magnification of × 200 (**A**). The experiment was repeated twice, each time in triplicate. Four or more random fields were photographed in each well, the area labelled with green and red fluorescence measured and the ratio of green-to-red fluorescence calculated. The averages for each experimental group and the between-group comparisons were calculated (**B**). Mean±s.e.m.; ^*^P<0.05 *vs* Dox; ^#^*P*<0.05 *vs* AN-88 and AN88+Dox.

The mitochondria play a critical role in cell death in response to stress signals. Opening of the mitochondrial permeability transition pore, results in the loss of Δ*Ψ*m, a step preceding cell death (Crow *et al*, 2004). We examined the effect of treatment with Dox, AN-7 and AN-88 alone or the combined treatments of AN-7 or AN-88 with Dox on Δ*Ψ*m. Using the fluorescent probe JC-1, the control cultures were stained dominantly with a spotted red colour, indicating normal Δ*Ψ*m (Figure 2). Two independent experiments were performed and quantified by the ImagePro Plus 5.1 software analysis. The ratio of green to red areas is proportional to the extent of reduction in Δ*Ψ*m (Figure 2B). Cultures of myocytes treated with Dox had the highest ratio of green to red, indicating that the Δ*Ψ*m collapsed and the cells were destined to undergo apoptosis. The myocyte cultures treated with AN-7 or Dox together with AN-7 were stained in red fluorescence that was indistinguishable from that of the untreated control culture, further supporting the protective role of AN-7 against Dox toxicity. AN-88, however, appeared to have a dual effect. When given as a single agent it augmented the ratio of green to red fluorescence suggesting cell damage, although substantially smaller than the damage caused by Dox. On the other hand, the combined treatment of Dox and AN-88 had the same effect on Δ*Ψ*m as AN-88 by itself, suggesting a certain degree of protection against Dox-induced dissipation of Δ*Ψ*m and retention of the AN-88 damaging capacity.

### Effect of Dox and the prodrugs on gene expression in cardiomyocytes

**Figure 3:**
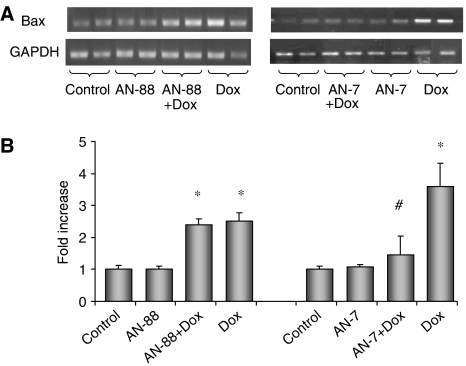
Effect of Dox and prodrugs on Bax expression in cardiomyocytes. Cardiomyocytes (6 × 10^6^) were seeded in 60-mm dishes and treated with 100 *μ*M AN-7 or 100 *μ*M AN-88 or 2 *μ*M Dox or the combination of Dox with the prodrugs as in Figure 1. Total RNA was isolated after 24 h of treatment and processed for semiquantitative RT–PCR analysis of Bax mRNA level and normalisation to the GAPDH mRNA level. (**A**) A representative gel of RT–PCR products. The variation within the duplicate of Dox treatment is caused by uneven loading as can be seen from the GAPDH that serves as a loading control. The ratio of Bax mRNA to GAPDH mRNA corrects for it. (**B**) A bar graph summarising the results of two independent experiments performed in duplicate. ^*^P<0.05 *vs* the untreated control; ^#^*P*<0.05 *vs* Dox.

Cardiomyocytes were treated with Dox (2 *μ*M), AN-7 and AN-88 (100 *μ*M) and their combination and the influence of the different treatments on expression of Bax, the proapoptotic gene, was examined by semiquantitative RT–PCR. Figure 3 presents mRNA levels of expression as detected by the relative intensity of ethidium bromide staining, quantified and normalised to GAPDH expression. Doxorubicin caused an increase in Bax expression that was lowered dramatically when the cells were co-treated with AN-7. However, after co-treatment of Dox with AN-88, no such decrease was observed. When cardiomyocytes were exposed to AN-7 or AN-88 as single agents, the expression of Bax did not change (Figure 3).

### Cardiomyocytes are protected by the elevation of endogenous formaldehyde

**Figure 4:**
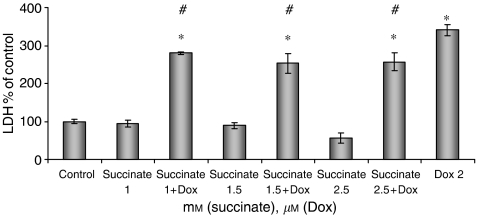
Succinate reduces Dox-induced damage in cultured cardiomyocytes. Cardiomyocytes (5 × 10^5^ cells well^−1^) were seeded in 24-well plates and treated with succinate (1, 1.5, 2 mM), Dox (2 *μ*M) and their combination. Lactate dehydrogenase activity in the growth medium was measured after 24 h as specified in Materials and Methods. Data summarise four independent experiments performed in triplicate. ^*^P<0.05 *vs* control; ^#^*P*<0.05 *vs* Dox.

Although all the investigated prodrugs release BA, the protection of cardiomyocytes against Dox-induced toxicity was exerted only by those releasing formaldehyde (AN-7, AN-1) and not by the analogous acetaldehyde-releasing ones (AN-88, AN-11). Since succinic acid, by inhibition of formaldehyde dehydrogenase (Cinti *et al*, 1976), increases the cellular endogenous formaldehyde level, we examined the ability of succinate to protect cardiomyocytes. Cells were treated with Dox (2 *μ*M), succinic acid (1, 1.5, 2.5 mM) and their combination. Treatment of the cells with succinic acid alone had no significant effect on LDH released into the culture media, yet when co-treated with Dox a statistically significant reduction in Dox toxicity was obtained (Figure 4). This finding strongly supports the pivotal role of formaldehyde in cardiomyocyte protection.

### Histone hyperacetylation

**Figure 5:**
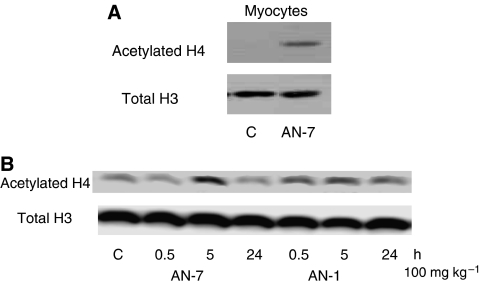
AN-7 induces histone hyperacetylation *in vitro* and *in vivo*. (**A**) *In vitro* acetylation in cardiomyocytes treated with AN-7 (100 *μ*M) for 4 h. The extracted histones were loaded 40 *μ*g protein lane^−1^. (**B**) Time course of histone acetylation in the heart of mice treated with 100 mg kg^−1^ AN-7 or AN-1 for the indicated duration (h). Histones were loaded 20 *μ*g protein lane^−1^. The protein samples were resolved on 15% SDS gels and subjected to Western blot analysis using the anti-acetylated H4 (lysine 12) and the anti H3 (as a loading control).

Previously, we have shown that in cancer cells the formaldehyde and the acetaldehyde releasing acyloxyalkyl-ester prodrugs increase histone acetylation (Nudelman *et al*, 2005; Rephaeli *et al*, 2005, 2006). Treatment of cardiomyocytes with 100 *μ*M AN-7 for 4 h also increased histone acetylation (Figure 5), indicating that the released BA was active as an inhibitor of HDAC in this cell type. When histones were isolated from the heart of mice treated with AN-7 or AN-1 (100 mg kg^−1^, p.o.), a transient increase in histone acetylation peaking at 5 h and diminishing after 24 h, was observed (Figure 5).

### Effect of Dox treatment as a single agent or in combination with the prodrugs on C57/BL mice

**Figure 6:**
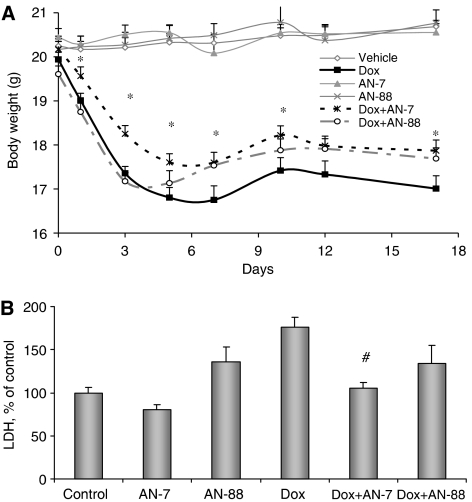
The effect of Dox and Dox combined with prodrugs on the body weight and serum LDH. Female C57/Bl mice were treated: once with: Dox 20 mg kg^−1^ (*n*=51), i.p.; AN-7 20 mg kg^−1^ (*n*=21), AN-88 20 mg kg^−1^ (*n*=11), vehicle (*n*=23) and the combination of AN-7+Dox (*n*=49) and AN-88+Dox (*n*=25), p.o., three times a week. (**A**) Body weight registration during 17 days. ^*^P<0.05, AN-7+Dox *vs* Dox. (**B**) Serum LDH (percent of the control) measured at termination of the experiment. ^*^P<0.05 *vs* control; ^#^*P*<0.05 *vs* Dox.

The increased histone acetylation in the heart suggested that the prodrugs reached the heart tissue where they released their metabolites. This observation provided the rationale that the prodrugs would be active *in vivo*. In three independent experiments, performed with C57/Bl mice, administration of a single i.p. dose of 20 mg kg^−1^ Dox caused a sharp decline in body weight during the first 7 days (Figure 6A). While mice treated with vehicle, p.o. AN-7 or p.o. AN-88 (20 mg kg^−1^) did not lose weight, a sharp decline in body weight in all the other Dox-treated groups was evident. This decline in body weight leveled off and even slightly reversed; nevertheless, it remained noticeably lower than that of mice not treated with Dox. In six out of the seven measurements, significant protection against weight-loss was seen in mice treated with Dox and AN-7. Co-treatment with Dox and AN-88 caused a similar decline in weight to Dox alone until day 5 when the body weight of the mice treated with AN-88 and Dox increased compared to those treated with only Dox. Mortality of the treated animals occurred on days 4–9 following Dox treatment and the subsequent increase in body weight may be attributed mainly to the death of the lowest-weighing mice in the group. In the Dox-treated group 45% of the mice died, while in the combined treatments of Dox with AN-7 or AN-88, 33 and 40% mortality occurred, respectively. When the mice were co-treated with AN-7 or AN-88 in addition to Dox, a lower percentage of mortality was noted. Although the overall decline in mortality in AN-7 and Dox cotreated mice was not statistically significant (*χ*^2^ test *P*=0.18, compared to treatment with Dox alone), in two of the three experiments, substantial protection by AN-7 was found (*χ*^2^ test *P*=0.003 and *P*=0.06 compared to Dox treatment). In the AN-88 and Dox cotreated groups, we did not observe significant protection compared to treatment with Dox alone. On termination of the experiments, blood was collected from representative surviving animals of each group and serum LDH activity was assessed. Over 70% increase in LDH activity was detected in the Dox, compared to the vehicle, treated group. While co-treatment of the mice with Dox and AN-7 prevented the rise in serum LDH, co-treatment with AN-88 did not (Figure 6B). These results are consistent with the weight-loss and the *in vitro* data. Since elevated LDH in serum indicates damage to organs in general and to the heart in particular, taken together with the weight-loss and the *in vitro* data, it implies that AN-7 protected against Dox toxicity *in vitro* and *in vivo*.

## DISCUSSION

Previously, we have reported that acyloxymethyl butyrate prodrugs possess anticancer activity and low toxicity, augmenting Dox toxicity toward cancer cells (Engel *et al*, 2006; Rephaeli *et al*, 2006). In this study we show, in primary cultures of cardiac myocytes and in C57/Bl mice, that these same prodrugs (AN-7 or AN-1) affect myocytes and animals in an opposite manner, namely, protecting them against Dox toxicity.

The effect of treatment with Dox and the prodrugs was tested initially on primary cultures of neonatal rat cardiac myocytes (Table 1). In reproducible experiments, only those prodrugs that release formaldehyde (AN-1 and AN-7) were effective in reducing the Dox damage as evident by the increased LDH leakage to the growth medium. The tested compounds encompassed two pairs (AN-7, AN-88 and AN-1, AN-11) that have the same chemical composition except that upon intracellular hydrolysis the former compound in each pair releases formaldehyde and the latter acetaldehyde (Table 1). From these data, it can be deduced that formaldehyde released together with BA endows cardiomyocyte protection against Dox, whereas acetaldehyde released together with BA does not. The possibility that formaldehyde alone endows protection or that acetaldehyde causes toxicity cannot be excluded. Succinic acid is known to increase the intracellular level of formaldehyde by inhibiting its oxidation to formic acid (Cinti *et al*, 1976; Kalasz, 2003). The observations that treatment of cells with acyloxymethyl esters results in measurable increase of formaldehyde concentration in the cell extract (Nudelman *et al*, 2005), and that treatment of the cardiomyocytes with succinic acid imparted protection (Figure 4), support the notion that formaldehyde participates in the protection activity. Taken together, it can be deduced that protection concurs with increased cellular formaldehyde. Along the same line, Burkhart *et al* (2006) have shown that a Dox-formaldehyde conjugate inhibited the growth of cancer cells better than that of cardiomyocytes while Dox displayed the opposite specificity.

The possibility that acetaldehyde harms cardiomyocytes was investigated in the context of alcohol-induced damage to the heart. It was suggested that acetaldehyde impairs cardiac function by promoting lipid peroxidation and oxidative damage (Aberle and Ren, 2003). However, in the LDH assay under the conditions employed, the acetaldehyde releasing prodrug AN-88, as a single agent and in combination with Dox, did not offer either toxicity or protection to the cardiomyocytes.

While the level of released LDH is indicative of plasma membrane permeabilisation, dissipation of mitochondrial membrane potential signifies that the cardiomyocytes undergo apoptosis (Crow *et al*, 2004; Gao *et al*, 2006). The changes in Δ*ψ*m evaluated by the JC-1 assay showed that treatment with Dox abolished Δ*Ψ*m while AN-7 had no effect and the combination of both prevented the dissipation. This is in agreement with the finding described above that AN-7 protects cardiomyocytes from Dox-induced leakage of cellular LDH. Unlike AN-7, AN-88 as a single agent caused dissipation of Δ*Ψ*m although to a lower extent than Dox. In the combination of AN-88 and Dox, the dissipation of Δ*Ψ*m was lower than Dox alone but similar to AN-88 as single agent. This implies that AN-88 has dual effect: on one hand, it causes damage to the cells and on the other, it attenuates Dox damage to the myocytes. With our current data, the identity of the protective or deleterious moiety cannot be resolved. In the LDH assay, the harmful effect of AN-88 on cardiomyocytes was not detected, possibly owing to the greater sensitivity of the membrane potential assay compared to the LDH leakage assay.

In addition to the reduction of Δ*Ψ*m, the observation that the expression of the proapoptotic gene Bax was upregulated by Dox and attenuated in the combined treatment of AN-7 and Dox, supports the ability of AN-7 to protect cardiomyocytes. While AN-7 and AN-88, as single agents, had no effect on Bax expression, AN-88 in combination with Dox did not moderate the Dox-enhanced Bax expression. The above indicates that AN-7 is superior to AN-88 not only as an anticancer agent (Nudelman *et al*, 2001), but also as a cardioprotective compound *in vitro*.

Potential clinical use of the protective effect of formaldehyde releasing prodrugs was tested in the frequently used Dox-induced toxicity mice model (Li *et al*, 2006; Lien *et al*, 2006). Three parameters confirmed the protective effects of AN-7 against Dox-induced toxicity: abolition of Dox-induced increase in the level of serum LDH, attenuation of Dox-induced loss in body weight and reduced mortality. Comparison between the *in vivo* effect of the combination of AN7 and Dox to that of AN-88 and Dox, further strengthens data obtained *in vitro* indicating that the formaldehyde released from AN-7 plays a major role in the protective effect. Histone acetylation seen in myocytes was also observed in hearts of mice treated with AN-7, indicating that the prodrug reached the heart and, as found in the myocytes, underwent metabolic hydrolysis in the heart (Figure 5). Unlike *in vitro*, where the protection is clearly of cardiomyocytes, *in vivo* the whole organism was targeted and the parameters measured were not restricted to the heart. Therefore, the prodrug-induced protection in the mice may involve other organs besides the heart and that will be subject to future study.

The molecular basis of AN-7 protection against Dox-induced cardiotoxicity could be explained by a number of different molecular mechanisms such as the protection of mitochondrial functions or reduced degradation of Dox to its toxic metabolite doxorubicinol (Mohamed *et al*, 2000; Minotti *et al*, 2001). Additional known damaging effects of Dox are the generation of ROS as well as the inhibition of the ROS detoxifying enzyme, glutathione peroxidase (Li *et al*, 2000). We postulate that cellular metabolic hydrolysis of the prodrugs results in the release of an HDAC inhibitory molecule (BA) and formaldehyde. The latter readily reacts with Dox to form a Dox-N=CH_2_ intermediate. Concurrently, the BA inhibits HDAC resulting in histone hyperacetylation and chromatin relaxation. This leads to greater accessibility of Dox-N=CH_2_ to the amino group on the guanine in GpC DNA sequences resulting in Dox-NH-CH_2_-NH-DNA (Dox–DNA adduct) formation (Cutts *et al*, 2005). The formed adducts sequester Dox to the nucleus and mitochondria reducing Dox availability to cytoplasmic degradation enzymes and redox-cycling that generate ROS. As a result, the adverse effects of the free cytoplasmic Dox are reduced.

Moreover, because of the intrinsic low toxicity of the prodrugs, their synergy with Dox would necessitate even lower doses of the Dox for cancer cell killing and will further reduce cardiotoxicity.

In contrast to the proliferating cancer cells, the mature cardiomyocytes do not divide, and consequently should be less debilitated by Dox–DNA adduct formation. In an unpublished preliminary study, a substantial increase in Dox–DNA adduct formation was detected by Southern hybridisation in the heart and muscle tissues of mice treated with the combination of Dox (15 mg kg^−1^) and formaldehyde releasing prodrug (200 mg kg^−1^). Adduct formation was not detected in these tissues of mice treated with Dox only. This initial observation supports our working hypothesis that the formation of Dox–DNA adducts diminishes Dox-induced damage to cardiomyocytes.

Our findings that AN-7 protects cardiomyocytes against damage caused by Dox and attenuates the Dox-induced morbidity in mice, is in contrast to our previously described synergism of the same drug combination in killing cancer cells (Engel *et al*, 2006; Rephaeli *et al*, 2006). The observations that a single molecule displays opposing biological roles, toward cancer *vs* normal myocardial cells, demonstrate diversity of activity tailored to particular cell types or tissues that is most desirable for antineoplastic agent and further support the potential AN-7 for cancer treatment. The molecular pathways underlying the cell-type-specific response are currently investigated by us.

## Figures and Tables

**Table 1:**
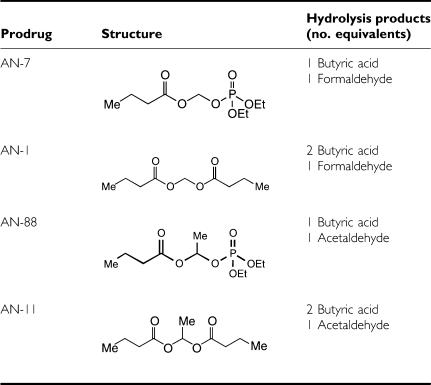
Structure and hydrolysis metabolites of the investigated prodrugs

**Table 2:** Primers and PCR conditions

**Gene**	**Primers (5′–3′)**	**Product size (bp)**	**Annealing T (°C)**	**Cycles**
Bax [I]	GCCCACCCAGCTCTGAACAGTCTGCTCGATCCTGGATGAAAC	71	63	32
Bax [II]	TGCAGAGGATGATTGCTGACGGAGGAAGTCCAGTGTCCAG	207	60	30
GAPDH	CCATGGAGAAGGCTGGGGCAAAGTTGTCATGGATGACC	195	59	20

GAPDH=glyceraldehyde-3-phosphate dehydrogenase.
